# Invasive Meningococcal Disease, 2011–2020, and Impact of the COVID-19 Pandemic, England

**DOI:** 10.3201/eid2709.204866

**Published:** 2021-09

**Authors:** Sathyavani Subbarao, Helen Campbell, Sonia Ribeiro, Stephen A. Clark, Jay Lucidarme, Mary Ramsay, Ray Borrow, Shamez Ladhani

**Affiliations:** Public Health England, London, UK (S. Subbarao, H. Campbell, S. Ribeiro, M. Ramsay, S. Ladhani);; Manchester Royal Infirmary, Manchester, UK (S.A. Clark, J. Lucidarme, R. Borrow);; St. George’s University of London, London (S. Ladhani)

**Keywords:** Neisseria meningitidis, meningococcal disease, meningococcal vaccines, bacterial co-infections, case fatality rate, SARS-CoV2, COVID-19, coronavirus, 2019 novel coronavirus disease, severe acute respiratory syndrome coronavirus 2, zoonoses, coronavirus disease, viruses, bacteria, England, United Kingdom

## Abstract

Invasive meningococcal disease incidence in England declined from 1.93/100,000 persons (1,016 cases) in 2010–11 to 0.95/100,000 (530 cases) in 2018–19 and 0.74/100,000 in 2019–20 (419 cases). During national lockdown for the coronavirus disease pandemic (April–August 2020), incidence was 75% lower than during April–August 2019.

*Neisseria meningitidis* is a major global cause of bacterial meningitis and septicemia (*1*). Six serogroups (A, B, C, W, X, Y) are responsible for most invasive meningococcal disease (IMD) cases (*1*). In the United Kingdom, implementation of serogroup C (MenC) meningococcal conjugate vaccine in 1999 led to sustained declines in MenC disease (*2*). In August 2015, an emergency adolescent MenACWY immunization program for persons 13–18 years of age and new university students was implemented to control a national outbreak of a hypervirulent MenW strain belonging to sequence type 11 clonal complex (MenW:cc11) (*3*). In September 2015, the United Kingdom became the first country to add a protein-based meningococcal B vaccine, 4CMenB, into the national infant immunization program (*4*). Both programs have reduced IMD caused by the respective vaccine serogroups (*5*). 

Since December 2019, the novel coronavirus (COVID-19) pandemic has led to major changes in the epidemiology of bacterial and viral infections worldwide (Brueggemann AB et al., unpub. data, https://www.medrxiv.org/content/10.1101/2020.11.18.20225029v1). We report IMD incidence in England during 2011–2020, including the impact of a national lockdown to control the spread of severe acute respiratory syndrome coronavirus 2 (SARS-CoV-2).

Public Health England (PHE) conducts national surveillance of IMD (*6*) and SARS-CoV-2 (*7*) in England. IMD incidence was highest, 1.93 cases/100,000 population (1,016 total cases), during the 2010–11 academic year (September–August) and declined to 1.15 cases /100,000 population for 2013–14 (617 cases) before increasing to 1.51 cases /100,000 population (825 cases) in 2015–16 (Figure). Adolescent MenACWY and infant 4CMenB immunization programs in 2015 led to additional annual declines in IMD incidence, to 0.95 cases /100,000 population (530 cases) in 2018–19 (incidence rate ratio [IRR] 0.63 [95% CI 0.56–0.70] for 2018–19 vs. 2015–16 ). Incidence further declined during the 2019–20 pandemic year (419 cases; 0.74 cases /100,000 population; IRR 0.49 [95% CI 0.44–0.56] for 2019–20 vs. 2015–16). IMD cases declined for all serogroups from 2015–16 to 2019–20: MenB by 38% (from 452 to 279 cases), MenC by 41% (41 to 24 cases), MenW by 68% (218 to 70 cases) and MenY by 66% (108 to 37 cases). 

**Figure:**
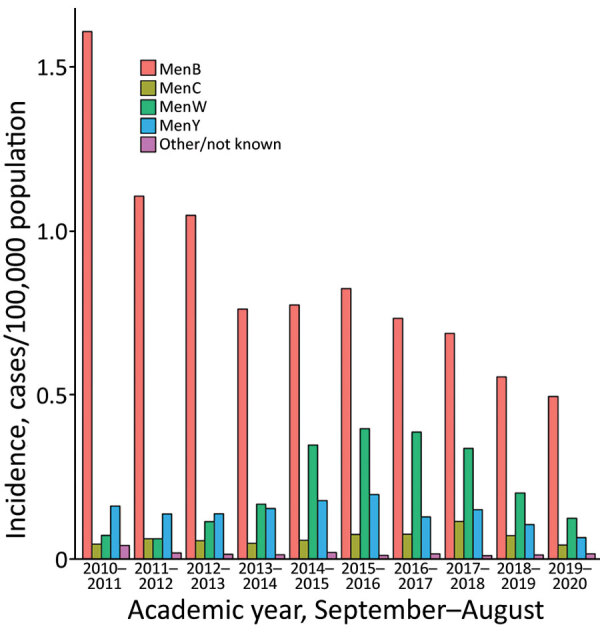
Cases of invasive meningococcal disease, by academic year, England, 2015–2020. Men, meningococcal conjugate vaccine (by serogroup).

IMD cases declined after the national COVID-19 lockdown on March 23, 2020, and remained low during April–August 2020. During 2018–19, PHE received 12,628 clinical samples from patients with suspected IMD; of these, 462 (4%) tested positive for *N. meningitidis*. These totals were 9,968 specimens, 401 (4%) positive, during 2019–20 (21% fewer cases). During April–August 2020, a total of 50 (1.8%) of 2,808 samples tested positive for *N. meningitidis*, compared with 134 (2.7%) of 5,025 samples during the same period in 2019 (p = 0.016). Combining culture-confirmed and PCR-confirmed cases, IMD incidence was 75% lower (IRR 0.25, 95% CI 0.18–0.35) during April–August 2020 than during April–August 2019 (Table). In contrast, IMD incidence during September 2019–March 2020 (the 7 months before national lockdown) was similar to that for September 2018–March 2019 (IRR 1.06, 95% CI 0.91–1.23). Declines were observed for all age groups and serogroups. During lockdown, compared with the same period during the previous year, MenB was overrepresented (33/45 [73%] vs. 104/179 [58%] cases), whereasMenW (5/45 [11%] vs. 42/179 [23%] cases) and MenY (0/45 [0%] vs. 16/179 [9%] cases) were underrepresented.

**Table:** Confirmed cases of meningococcal disease during April–August 2019 and April–August 2020, England*

Category	April–Aug 2019, no. (%)	April–Aug 2020, no. (%)	RR (95% CI)
Group			
Total (N)	179	45	0.25 (0.18–0.35)
MenB	104 (58)	33 (73)	0.32 (0.21–0.47)
MenC	14 (8)	5 (11)	0.36 (0.13–0.99)
MenW	42 (23)	5 (11)	0.12 (0.05–0.30)
MenY	16 (9)	0	0
Other	3 (2)	2 (4)	0.67 (0.11–3.97)
Age group, y			
Total	179	45	0.25 (0.18–0.35)
<5	39 (22)	17 (38)	0.44 (0.25–0.78)
5–14	20 (11)	4 (9)	0.2 (0.07–0.58)
15–24	24 (13)	4 (9)	0.17 (0.06–0.48)
25–64	53 (30)	14 (31)	0.26 (0.15–0.47)
>65	43 (24)	6 (13)	0.14 (0.06–0.32)

*The numbers of typeable strains during the specified time frame by age group are shown. RR, relative risk.

A total of 45 IMD cases were diagnosed during April–August 2020. The median age of patients was 67 (interquartile range 20–85) years. Linkage with national SARS-CoV-2 data identified 2 patients with IMD who were also positive for SARS-CoV-2 by reverse transcription PCR; both were <90 days of age with late-onset MenB meningitis, and 1 died. Meningitis (with or without septicemia) was proportionally more frequent during the lockdown months compared with the same period in 2019 (27/45 [60%] v. 71/179 [39.7%] cases; p = 0.014). Three (6.7%) of the 45 patients died within 28 days of diagnosis: the infant with co-infection, an adult with MenB meningitis, and an older adult with MenB septicemia.

Limitations of our study include limited clinical data collected for IMD cases. Cases and case-fatality rates during the lockdown period might also be underestimated if some patients died of IMD at home because they did not seek medical help earlier as a result of the stay at home messaging during lockdown.

In summary, IMD incidence in England has been declining since the early 2000s (*8*) because of the MenC immunization program and natural trends in MenB disease and further declined because of 2 new meningococcal immunization programs. National lockdown in March 2020 led to a 75% reduction in cases compared with the same period in the previous year, overrepresented MenB cases. Declines in IMD cases after national lockdown were also reported in France (*9*), which is reassuring because viral infections are known to precede IMD; therefore, SARS-CoV-2 could potentially have increased the risk of secondary bacterial infections. Our findings do not support wider vaccination against IMD during the COVID-19 pandemic.

Appendix Additional information on invasive meningococcal disease in England, 2011–2020.
