# Supplementary material for: Invasive Meningococcal Disease, 2011–2020, and Impact of the COVID-19 Pandemic, England
Source: Emerg Infect Dis. 2021 Sep;27(9):2495–7. doi: 10.3201/eid2709.204866 (PMC8386802; doi:10.3201/eid2709.204866)
Supplement: Appendix — Additional information on invasive meningococcal disease in England, 2011–2020. [file 20-4866-Techapp-s1.pdf]

Article DOI: https://doi.org/10.3201/eid2709.204866

Invasive Meningococcal Disease, 2011–2020, and Impact of the COVID-19 Pandemic, England

Appendix

Appendix Table. Demographic, clinical, and microbiological information comparing cases of meningococcal disease during April–August 2019 and 2020 using χ^2 test of independence

Characteristic	April–Aug 2019 n (%)	April–Aug 2020 n (%)	χ^2 test of independence p value
Total	179 (100%)	45 (100%)	
Sex			0.301
Female	92 (51%)	27 (60%)	
Male	87 (49%)	18 (40%)	
Age group, y			0.175
<5	39 (22%)	17(38%)	
5–14	20 (11%)	4 (9%)	
15–24	24 (13%)	4 (9%)	
25–64	53 (30%)	14 (31%)	
>65	43 (24%)	6 (13%)	
Serogroup			0.046
MenB	104 (58%)	33 (73%)	
MenC	14 (8%)	5 (11%)	
MenW	42 (23%)	5 (11%)	
MenY	16 (9%)	0 (0%)	
Other/not known	3 (2%)	2 (4%)	
Clincial presentation			0.097
Meningitis	27 (15%)	13 (29%)	
Septicemia	87 (49%)	15 (33%)	
Meningitis and septicemia	44 (25%)	14 (31%)	
Pneumonia	5 (3%)	0 (0%)	
Other source	16 (9%)	3 (7%)	
Identification			0.028
Culture positive	76 (42%)	12 (27%)	
PCR positive	61 (34%)	25(56%)	
Culture/PCR positive	42 (23%)	8 (18%)	
Death within 28 d	13 (7%)	3 (7%)	0.89

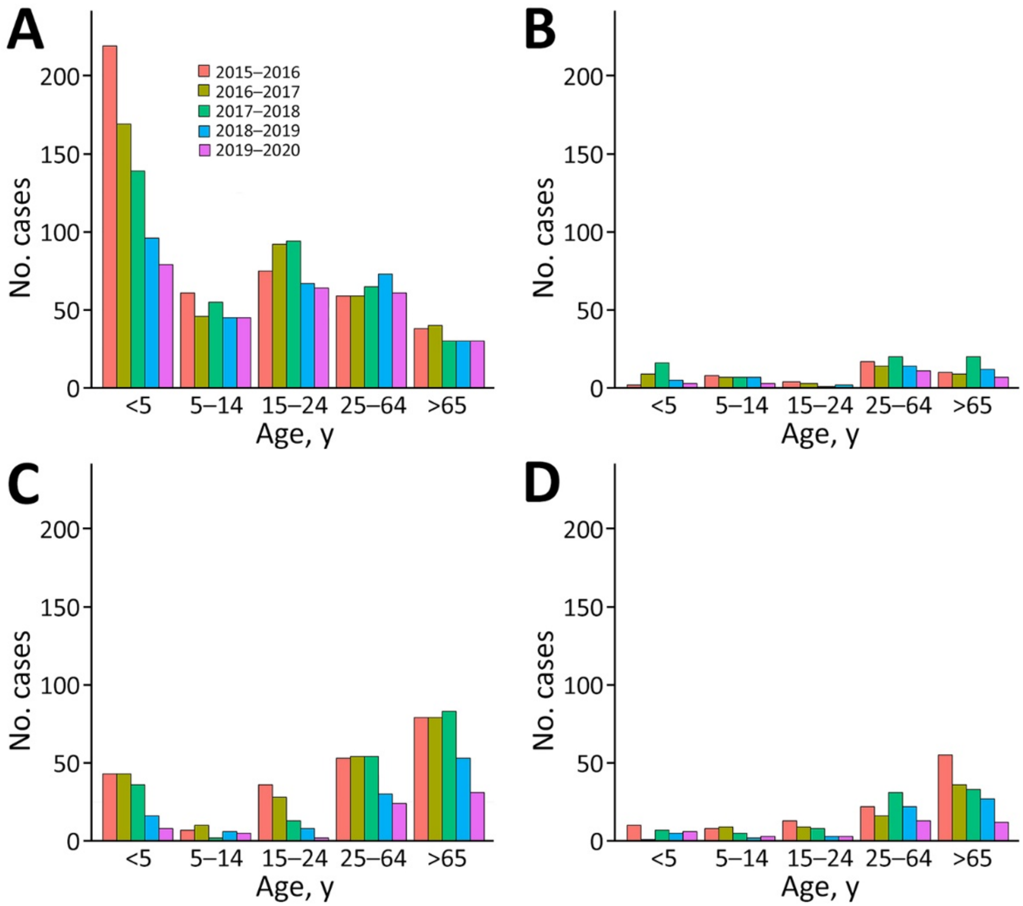

Appendix Figure 1. Annual number of cases of invasive meningococcal disease, England, 2015–2020: A) MenB; B) MenC; C) MenW; D) MenY.

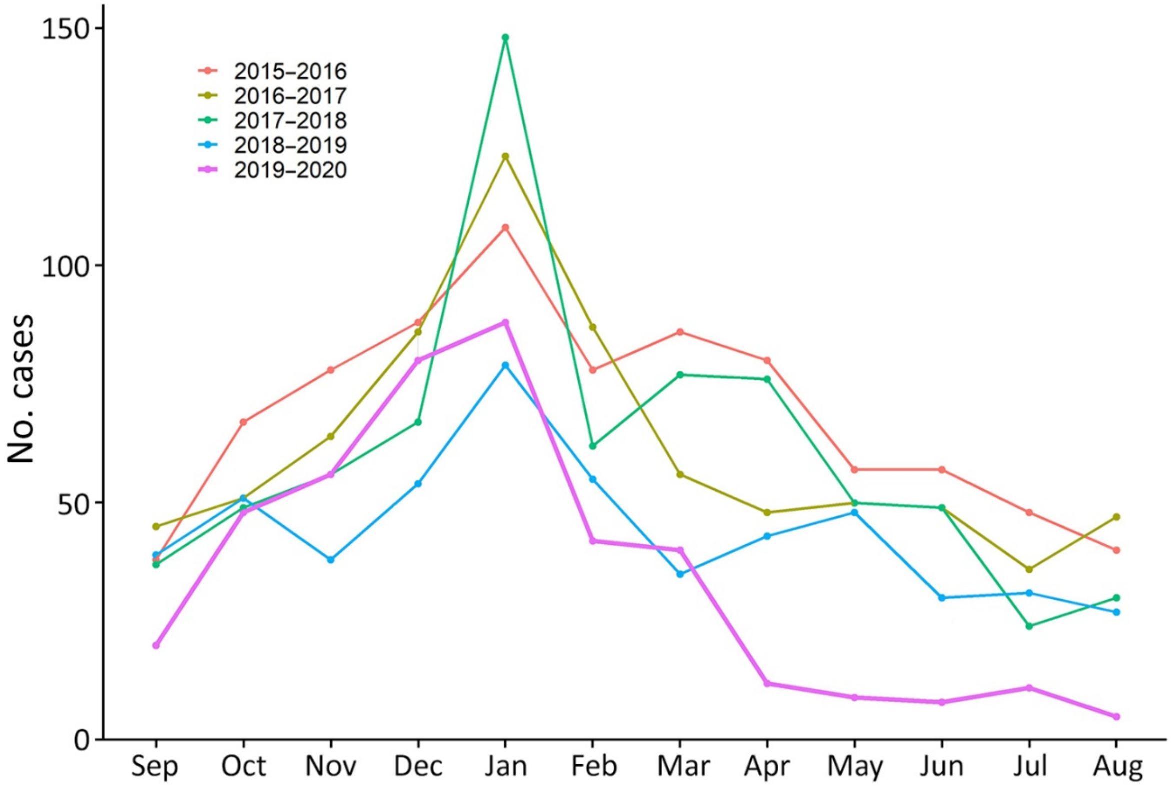

Appendix Figure 2. Total numbers of cases of invasive meningococcal disease, England, 2015–2020.
